# Wetland Sediments Host Diverse Microbial Taxa Capable of Cycling Alcohols

**DOI:** 10.1128/AEM.00189-19

**Published:** 2019-05-30

**Authors:** Paula Dalcin Martins, Jeroen Frank, Hugh Mitchell, Lye Meng Markillie, Michael J. Wilkins

**Affiliations:** a Department of Microbiology, Radboud University Nijmegen, Nijmegen, The Netherlands; b Pacific Northwest National Laboratory, Richland, Washington, USA; c Department of Soil and Crop Sciences, Colorado State University, Fort Collins, Colorado, USA; University of Georgia

**Keywords:** alcohol cycling, fermentation, organic matter degradation, sediments, wetlands

## Abstract

Alcohols are commonly derived from the degradation of organic matter and yet are rarely measured in environmental samples. Wetlands in the Prairie Pothole Region (PPR) support extremely high methane emissions and the highest sulfate reduction rates reported to date, likely contributing to a significant proportion of organic matter mineralization in this system. While ethanol and isopropanol concentrations up to 4 to 5 mM in PPR wetland pore fluids have been implicated in sustaining these high rates of microbial activity, the mechanisms that support alcohol cycling in this ecosystem are poorly understood. We leveraged metagenomic and transcriptomic tools to identify genes, pathways, and microorganisms potentially accounting for alcohol cycling in PPR wetlands. Phylogenetic analyses revealed diverse alcohol dehydrogenases and putative substrates. Alcohol dehydrogenase and aldehyde dehydrogenase genes were included in 62 metagenome-assembled genomes (MAGs) affiliated with 16 phyla. The most frequently encoded pathway (in 30 MAGs) potentially accounting for alcohol production was a Pyrococcus furiosus-like fermentation which can involve pyruvate:ferredoxin oxidoreductase (PFOR). Transcripts for 93 of 137 PFOR genes in these MAGs were detected, as well as for 158 of 243 alcohol dehydrogenase genes retrieved from these same MAGs. Mixed acid fermentation and heterofermentative lactate fermentation were also frequently encoded. Finally, we identified 19 novel putative isopropanol dehydrogenases in 15 MAGs affiliated with *Proteobacteria*, *Acidobacteria*, *Chloroflexi*, *Planctomycetes*, *Ignavibacteriae*, *Thaumarchaeota*, and the candidate divisions KSB1 and *Rokubacteria*. We conclude that diverse microorganisms may use uncommon and potentially novel pathways to produce ethanol and isopropanol in PPR wetland sediments.

**IMPORTANCE** Understanding patterns of organic matter degradation in wetlands is essential for identifying the substrates and mechanisms supporting greenhouse gas production and emissions from wetlands, the main natural source of methane in the atmosphere. Alcohols are common fermentation products but are poorly studied as key intermediates in organic matter degradation in wetlands. By investigating genes, pathways, and microorganisms potentially accounting for the high concentrations of ethanol and isopropanol measured in Prairie Pothole wetland sediments, this work advanced our understanding of alcohol fermentations in wetlands linked to extremely high greenhouse gas emissions. Moreover, the novel alcohol dehydrogenases and microbial taxa potentially involved in alcohol metabolism may serve biotechnological efforts in bioengineering commercially valuable alcohol production and in the discovery of novel isopropanol producers or isopropanol fermentation pathways.

## Abstract (precis)

Understanding patterns of organic matter degradation in wetlands is essential for identifying the substrates and mechanisms supporting greenhouse gas production and emissions from wetlands, the main natural source of methane in the atmosphere. Alcohols are common fermentation products but are poorly studied as key intermediates in organic matter degradation in wetlands. By investigating genes, pathways, and microorganisms potentially accounting for the high concentrations of ethanol and isopropanol measured in Prairie Pothole wetland sediments, this work advanced our understanding of alcohol fermentations in wetlands linked to extremely high greenhouse gas emissions. Moreover, the novel alcohol dehydrogenases and microbial taxa potentially involved in alcohol metabolism may serve biotechnological efforts in bioengineering commercially valuable alcohol production and in the discovery of novel isopropanol producers or isopropanol fermentation pathways.

## INTRODUCTION

Fermentation is a primary mode of organic matter degradation. Fermentative pathways can both result in carbon mineralization and generate substrates that fuel anaerobic respiration, contributing to methane (CH_4_) and carbon dioxide (CO_2_) emissions. The relevance of these interconnected processes in soils and sediments has been previously reported across a range of ecosystems. For example, Wrighton et al. (1) suggested that fermentation was a major route of carbon turnover in a shallow alluvial aquifer, resulting in the generation of a range of labile substrates, including hydrogen, ethanol, formate, acetate, lactate, and butyrate. In this system these intermediates were inferred to support nitrate, sulfate, and iron reduction. In boreal fens, the fermenter community differed between high-CH_4_- and high-CO_2_-producing peat slurry incubations, indicating that various fermentative pathways may impact the CO_2_/CH_4_ ratio in greenhouse gas emissions (2). In marine sediments, temperature perturbation experiments revealed a close coupling between fermentation and sulfate reduction (3), while iron reduction and methanogenesis were inferred to be supported by fermentation products in Arctic tundra soils (4).

Although alcohols—particularly ethanol, but also 1-propanol, isopropanol (2-propanol), and butanol—are common fermentation products, their role in stimulating sedimentary carbon cycling has received little attention. Indeed, knowledge gaps associated with the magnitude and fate of ethanol produced in wetlands represent a fundamental constraint in estimating global carbon budgets (5). Technical issues have frequently precluded the measurement of alcohols in complex environmental samples. For instance, pore water samples for proton-nuclear magnetic resonance (^1^H-NMR) are often concentrated using alcohols, a process which prohibits the measurement of natural alcohol abundances in the sample (6). However, there are some instances of alcohols being measured in sedimentary samples; in 2005, Metje and Frenzel reported ethanol concentrations of up to 10.5 mM in methanogenic peat soil incubations (7), while Zhuang et al. developed pretreatment techniques to measure ethanol and methanol using gas chromatography in marine sediment pore waters, reporting ethanol concentrations that ranged from 3 to 62 µM (8). The same technique was used to measure concentrations of ethanol ranging from 11 to 2,535 nM in freshwater sediments (9).

The Prairie Pothole region (PPR) of North America is the tenth largest wetland ecosystem in the world (10). Using nonconcentrated sediment pore waters, ethanol and isopropanol concentrations of up to 4 to 5 mM have been measured using ^1^H-NMR in PPR wetland sediments (11), suggesting that fermentation may play a key role in organic matter degradation into these alcohols. This ecosystem is carbon rich, with pore fluid dissolved organic carbon concentrations reaching ∼180 mg/liter (12). In addition, extremely high methane fluxes (∼160 mg CH_4_/m^2^/h) and the highest sulfate reduction rates ever reported to date (∼22 µmol/cm^3^/day) have been measured in PPR wetlands (11). The depletion of alcohols during a period of high sulfate reduction, as well as the identification of candidate sulfate-reducing bacterium genomes encoding alcohol dehydrogenases, suggested a possible role for these substrates in driving sulfate reduction in this system. Moreover, the detection of F420-dependent alcohol dehydrogenases and *mcrA* genes affiliated with alcohol-utilizing *Methanofollis* species indicated that methanogenesis may also be directly supported by these fermentation products (13). Despite the potential importance of alcohols in supporting biogeochemical activity in PPR sediments, the microbial members and the pathways responsible for alcohol fermentation in this system remain unknown.

Here, we used metagenomics and metatranscriptomics to investigate putative alcohol-cycling microorganisms in PPR wetland sediments using sequencing data obtained for samples previously analyzed (11, 13). We have examined genome-encoded alcohol dehydrogenases and pathways that could result in ethanol and isopropanol production in Prairie Pothole wetlands. Our results indicate that known and novel pathways for alcohol cycling are active across phylogenetically diverse microbial groups in this ecosystem and that a variety of novel alcohol dehydrogenases have yet to be characterized. These results have both environmental relevance—in the context of carbon cycling and greenhouse gas emissions—and industrial importance, given the decades of efforts in engineering microorganisms for the production of these alcohols (14, 15).

## RESULTS

### Putative alcohol-cycling microorganisms are phylogenetically diverse and encode a variety of alcohol dehydrogenases.

Depth-resolved metagenomic data sets were obtained from sediments in two characteristic wetlands near Jamestown, ND, and processed as previously described (13). These wetlands are rich in dissolved organic carbon (12) and sulfur compounds (16, 17) due to the local hydrological regime and the underlying pyrite-rich glacial till (18). Dynamic shifts in redox conditions occur in these wetlands due to annual and seasonal rainfall and temperature changes, storm events that transport agricultural runoff into the wetlands, and prairie winds, which can mix and oxygenate the shallow (1- to 3-m) water column (19). Samples analyzed in this study had been previously characterized for pore water concentrations of methane, sulfate, sulfide, ethanol, methanol, isopropanol, and acetate, as well as for sulfate reduction rates, dissolved organic carbon compounds, and 16S rRNA gene-based microbial communities (11). Remarkably, extremely high ethanol and isopropanol concentrations (up to 4 to 5 mM), methane concentrations (up to 6 mM), and sulfate reduction rates (up to 22 µmol/cm^3^/day) were measured in these sediments, with substrates depleted from spring to summer when methane emissions and sulfate reduction rates were highest (11).

**FIG 1:**
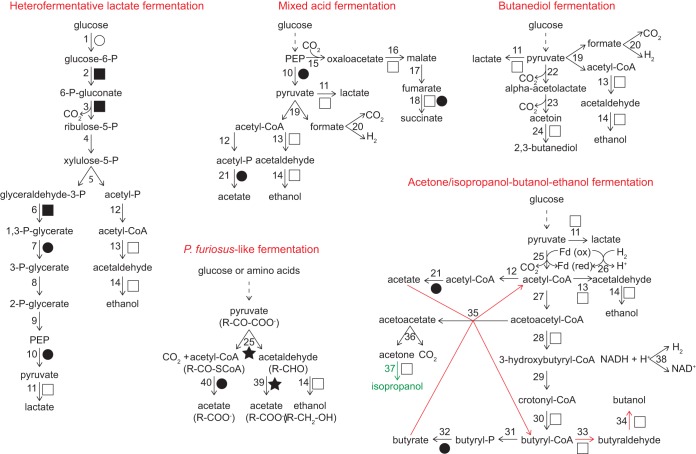
Simplified summary of investigated pathways for ethanol and isopropanol production. ATP-generating reactions are indicated by closed circles, and ATP-consuming reactions are indicated by open circles. NAD(P)H-generating reactions are indicated by closed squares. NAD(P)H-consuming reactions are indicated by open squares. Reduced ferredoxin [Fd(red)]-generating reactions are indicated by closed stars. Oxidized ferredoxin [Fd(ox)]-generating reactions are indicated by open stars. Solid arrows indicate the written reaction, while dashed arrows indicate a series of reactions not shown. In acetone/isopropanol-butanol-ethanol fermentation, red arrows indicate solventogenic phase reactions, and the green arrow and substrate indicate an additional reaction in the isopropanol-producing variation. In the Pyrococcus furiosus-like fermentation, “R-” indicates the radical in the molecule. Enzymes are numbered as follows: 1, hexokinase; 2, glucose-6-P dehydrogenase; 3, 6-P-gluconate dehydrogenase; 4, ribulose-5-P epimerase; 5, phosphoketolase; 6, glyceraldehyde-3-P dehydrogenase; 7, 3-P-glycerate kinase; 8, 3-P-glycerate mutase; 9, enolase; 10, pyruvate kinase; 11, lactate dehydrogenase; 12, phosphotransacetylase; 13, aldehyde dehydrogenase; 14, alcohol dehydrogenase; 15, phosphoenolpyruvate (PEP) carboxylase; 16, malate dehydrogenase; 17, fumarase; 18, succinate dehydrogenase; 19, pyruvate-formate lyase; 20, formate-hydrogen lyase; 21, acetate kinase; 22, alpha-acetolactate synthase; 23, alpha-acetolactatedecarboxylase; 24, 2,3-butanediol dehydrogenase; 25, PFOR, IFOR, OGFOR, or OIFOR; 26, ferredoxin hydrogenase; 27, acetyl-CoA acetyltransferase; 28, hydroxybutyryl-CoA dehydrogenase; 29, crotonase; 30, butyryl-CoA dehydrogenase; 31, phosphotransbutyrylase; 32, butyrate kinase; 33, butyraldehyde dehydrogenase; 34, butanol dehydrogenase; 35, acetoacetyl-CoA:acetate/butyrate:CoA transferase; 36, acetoacetate decarboxylase; 37, isopropanol dehydrogenase; 38, hydrogen dehydrogenase; 39, aldehyde:ferredoxin oxidoreductase; 40, acetyl-CoA synthetase. Fermentations are not balanced, and reversible reactions are not indicated.

Metagenome-assembled genomes (MAGs) were screened for the presence of both an aldehyde dehydrogenase and an alcohol dehydrogenase (ADH) with potential for primary or secondary alcohol fermentation or oxidation, which excluded short-chain, aryl, and polyvinyl ADHs, as well as choline, sugar-alcohol, and phosphonate catabolism-related ADHs. Of 449 MAGs recovered from our metagenomic data sets, 62 had at least one gene encoding each enzyme and estimated contamination levels of less than 13% and thus were selected for further analyses. Known pathways for alcohol fermentation (Fig. 1) were investigated in order to determine the potential for ethanol and isopropanol production.

**FIG 2:**
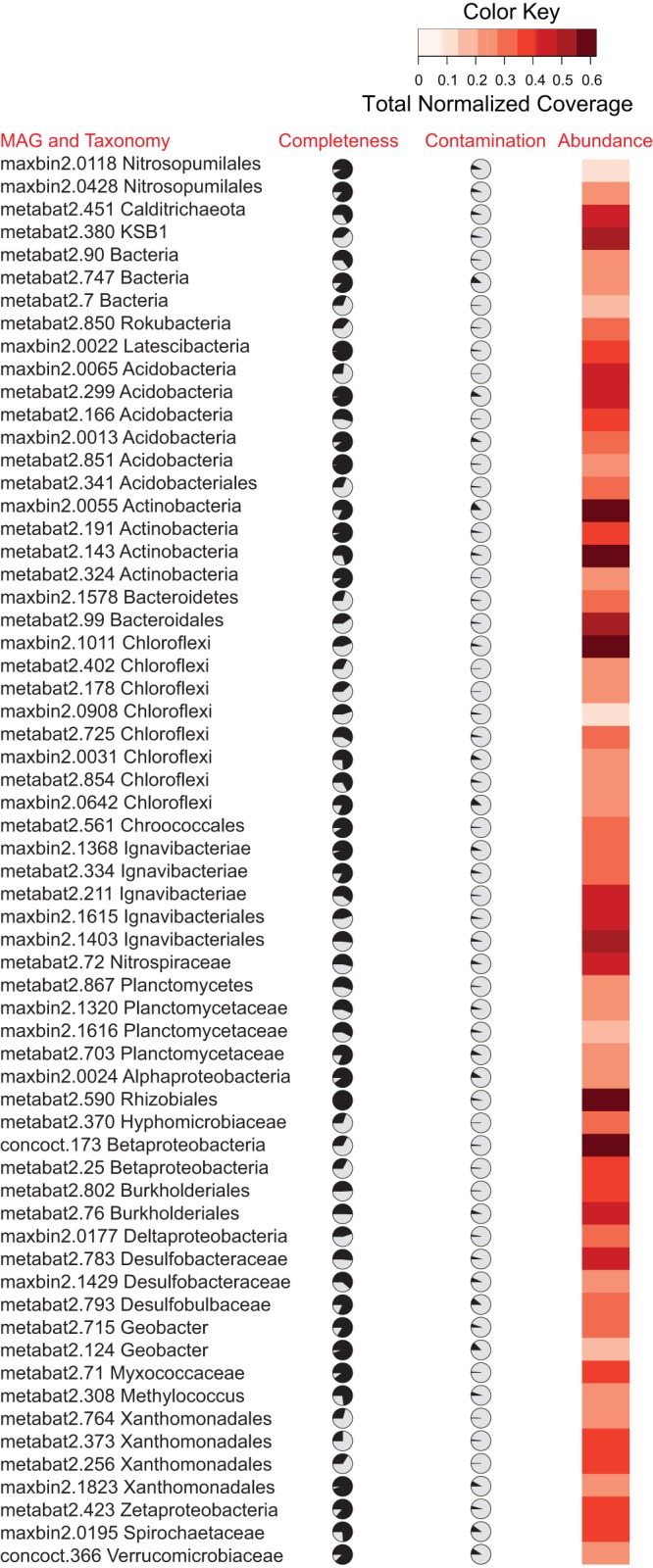
Overview of metagenome-assembled genomes selected for this study. MAG name, taxonomy, completeness, contamination, and abundance are provided. Taxonomy was inferred with CheckM and phylogenetic analyses of RpsC sequences. Abundance is expressed as total normalized coverage (across all metagenomes) per Gbp of metagenome (see Materials and Methods for details).

An overview of the MAGs selected for this study is provided in Fig. 2. Detailed MAG information on genome and investigated genes is provided in Table S1 in the supplemental material. The selected MAGs had abundances (Table S1) varying between 0.12 and 0.62 total coverage normalized per Gbp of metagenome (average, 0.34; median, 0.31), which is well within general trends observed for the entire 449-MAG data set (minimum coverage of 0.004 and maximum of 2.5, with an average of 0.26 and a median of 0.25).

**FIG 3:**
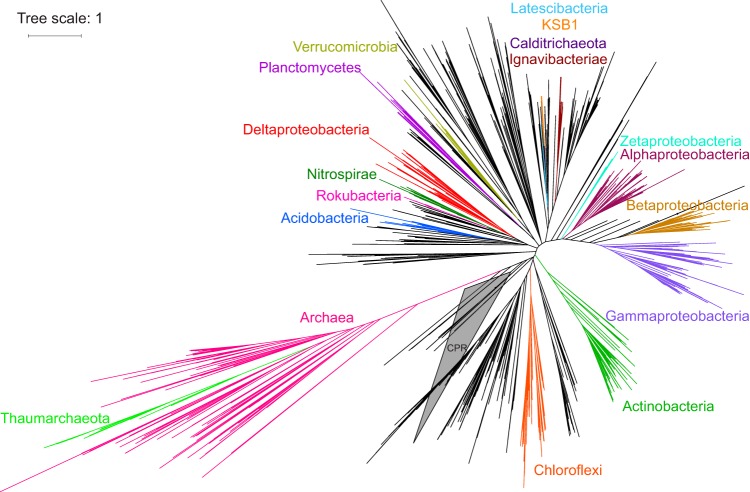
Phylogeny of alcohol-cycling microorganisms spanning the archaeal and bacterial tree of life based on reference and MAG-retrieved RpsC sequences. Binned sequences were present in the color-coded clades. Only taxonomic groups containing binned RpsC sequences are labeled; however, some MAGs were lacking the *rpsC* gene (27/62). In these instances, taxonomy was inferred solely with CheckM (such MAGs are absent from this tree). Taxonomic groups are labeled by the branch. CPR, candidate phyla radiation (collapsed clade).

The selected MAGs representing candidate alcohol-cycling microorganisms spanned 16 phylum-level taxonomic groups (Fig. 3): *Proteobacteria* (*n* = 20 MAGs), *Chloroflexi* (*n* = 8), *Acidobacteria* (*n* = 6), *Ignavibacteriae* (*n* = 5), *Actinobacteria* (*n* = 4), *Planctomycetes* (*n* = 4), *Bacteroidetes* (*n* = 2), *Thaumarchaeota* (*n* = 2), *Cyanobacteria* (*n* = 1), *Nitrospirae* (*n* = 1), *Spirochaetes* (*n* = 1), *Verrucomicrobia* (*n* = 1), *Calditrichaeota* (*n* = 1), and candidate phyla KSB1 (*n* = 1), *Rokubacteria* (*n* = 1), and *Latescibacteria* (*n* = 1). Three bacterial MAGs were unclassified.

**FIG 4:**
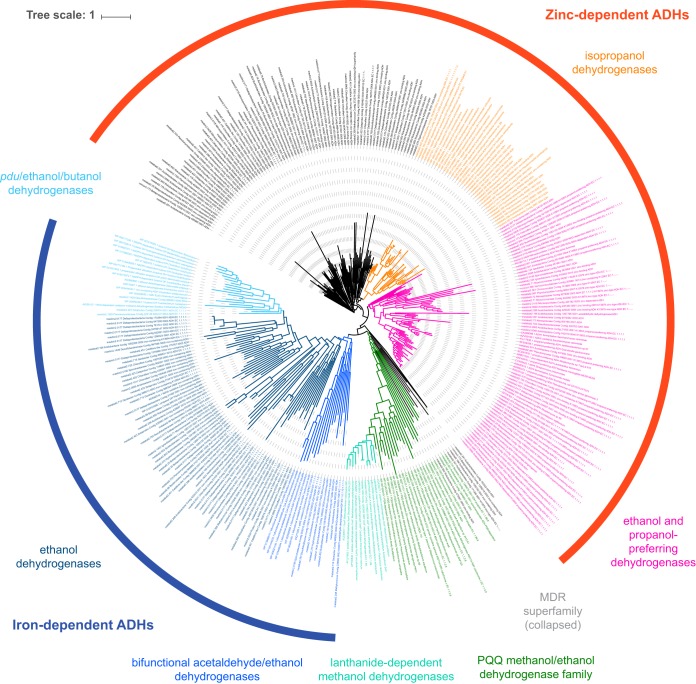
Alcohol dehydrogenase phylogenetic tree. Zinc-type and iron-type ADHs are indicated by the outside semicircles in red and blue, respectively. Of 415 sequences in total, 76 did not cluster with any reference sequences (black color within zinc-type zone), 36 were classified as isopropanol dehydrogenases (orange), 75 were classified as propanol/ethanol dehydrogenases (pink), 91 were NADH:quinone oxidoreductases and other medium-chain reductase family members (collapsed branch), 38 formed a cluster of mostly PQQ-type ADHs (green shades), and 99 formed a cluster of mostly ethanol dehydrogenases (blue shades). Abbreviations: ADH, alcohol dehydrogenase; MDR, medium-chain reductases; PQQ, pyrroloquinoline quinone; *pdu*, propanediol utilization alcohol dehydrogenase gene. An interactive online version of this tree (ADHs_in_62_MAGs_and_refs.tree) is available at https://itol.embl.de/shared/pdalcin.

A wide variety of alcohol dehydrogenases with potential for alcohol fermentation or oxidation were recovered from PPR draft genomes, varying in number between 1 and 30 per MAG. Overall, 366 ADHs were identified, with 334 being at least 100 amino acids long. Phylogenetic analyses of these sequences (Fig. 4) indicated that ADHs formed clusters primarily based on cofactor, with a major segregation between iron-, zinc-, and pyrroloquinoline quinone (PQQ)-type ADHs, and secondarily based on putative substrate preference. A monophyletic cluster of 91 poorly annotated ADHs was determined to contain NADH:quinone oxidoreductases and uncharacterized medium-chain dehydrogenase superfamily members (collapsed branch in Fig. 4), which excluded the corresponding genes from further analyses. Of the remaining 243 ADH genes, 158 were inferred to be expressed within the microbial community via metatranscriptomic data (Table S2).

Reference isopropanol dehydrogenases included in the phylogenetic analysis formed a monophyletic group with 36 sequences in total, allowing the identification of 19 binned ADHs as putative isopropanol dehydrogenases. These sequences belonged to 15 MAGs affiliated with the betaproteobacterial order *Burkholderiales*, the alphaproteobacterial family *Hyphomicrobiaceae*, the phyla *Acidobacteria*, *Ignavibacteriae*, *Chloroflexi*, *Planctomycetes*, and *Thaumarchaeota*, candidate divisions KSB1 and *Rokubacteria*, and one unclassified bacterial genome. Of these 19 putative isopropanol dehydrogenase genes, 14 were detected in metatranscriptomic data (Table S2).

Another monophyletic group contained mostly propanol-preferring ADHs (71 sequences) plus only four ethanol-preferring reference ADHs (both fermentative and oxidative ADHs from Saccharomyces cerevisiae, ADH-I from Streptococcus pneumoniae, and the fermentative ADH-I from Zymomonas mobilis). Similarly, PQQ-type methanol/ethanol dehydrogenases (34 sequences) also formed a monophyletic group that also contained two zinc-type and two iron-type ADHs. Interestingly, eleven lanthanide-dependent methanol dehydrogenases (including two unbinned sequences from our metagenomes annotated as XoxF) formed a monophyletic group within the PQQ-type ADH branch (Fig. 4).

Finally, 99 sequences formed a branch of mostly inferred ethanol dehydrogenases. Bifunctional acetaldehyde/ethanol dehydrogenases formed two monophyletic groups; a subbranch of sequences almost exclusively identified in this work contained only two reference sequences (the propanediol utilization propanol dehydrogenase PduQ from Enterococcus faecalis and the oxidative ADH-II from Zymomonas mobilis), while a second subbranch contained only 3 sequences from this study, and 16 reference sequences (9 butanol dehydrogenases, 4 ethanol dehydrogenases, 2 propanediol utilization propanol dehydrogenases, and 1 methanol dehydrogenase), with 4 of these reference sequences obtained from *Clostridium* species.

### Known, unusual, and novel pathways may be used for ethanol and isopropanol fermentation in prairie pothole wetland sediments.

**FIG 5:**
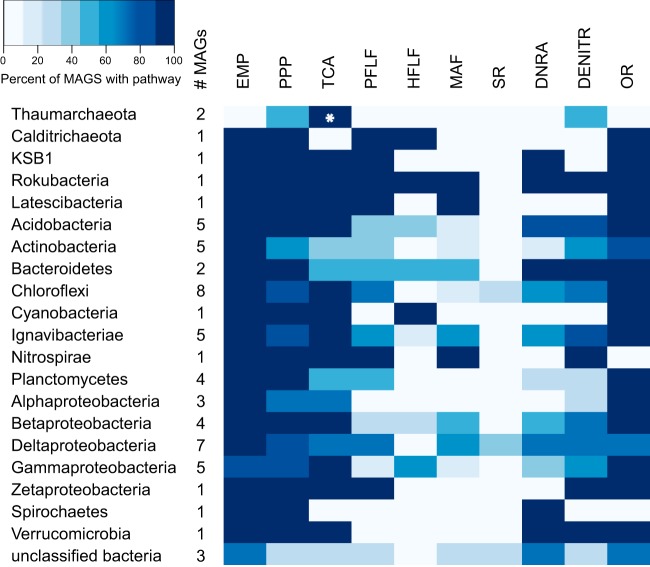
Summary of metabolic potential in MAGs by taxa. The shades of blue indicate the percentages of MAGs that encoded the potential for each pathway. The number of MAGs in each taxa is indicated under “#MAGs.” Abbreviations: EMP, Embden-Meyerhof-Parnas glycolysis; PPP, pentose phosphate pathway; PDH, pyruvate dehydrogenase complex; TCA, tricarboxylic acid cycle; PFLP, P. furiosus-like fermentation; HFLF, heterofermentative lactate fermentation; MAF, mixed acid fermentation; SR, sulfate reduction; DNRA, dissimilatory nitrate reduction to ammonium; DENITR, partial denitrification; OR, oxygen reduction. The asterisk (*) indicates an incomplete TCA cycle.

In this study, we additionally analyzed central carbon metabolism pathways in the context of alcohol fermentations. Respiratory processes were also examined in order to determine whether MAGs could represent facultative or obligate fermenters (Table S1). Investigated fermentation pathways are represented in Fig. 1, and the MAG genomic potential is summarized by taxa in Fig. 5 (with a detailed gene presence/absence report provided in Table S1). Detailed descriptions of inferred metabolisms in each MAG are presented in the supplemental material. The majority of the MAGs (59 of 62) encoded sugar utilization systems, the machinery for Embden-Meyerhof-Parnas (EMP) glycolysis (58 of 62), and the pentose phosphate pathway (52 of 62). Moreover, 50 of the MAGs had a pyruvate dehydrogenase complex, and 49 contained tricarboxylic acid (TCA) cycle genes (2 *Thaumarchaeota* MAGs contained an incomplete TCA cycle). Only one MAG encoded the Entner-Doudoroff pathway.

The potential for alcohol fermentation was encoded across a range of pathways. Overall, 42 of 62 MAGs had potential for at least one of the fermentative pathways investigated, although all encoded at least one acetaldehyde dehydrogenase and one alcohol dehydrogenase, suggesting that novel pathways may exist in these microorganisms. P. furiosus-like fermentation was the most frequent fermentation pathway present in our MAGs (30 of 62), with 68% of the pyruvate:ferredoxin oxidoreductase (PFOR) genes in these MAGs identified in metatranscriptomic data (Table S3). In total, 48 MAGs encoded PFOR; 46, 2-oxoglutarate:ferredoxin oxidoreductase (OGFOR); 29, indolepyruvate:ferredoxin oxidoreductase (IFOR); 3, 2-oxoisovalerate:ferredoxin oxidoreductase (OIFOR); 49, acetyl coenzyme A synthetase (ACS), 22, aldehyde:ferredoxin oxidoreductase (AFOR); and 30, ferredoxin:NADP oxidoreductase. In addition, 18 MAGs contained the functional potential for mixed acid fermentation, and 11 MAGs for heterofermentative lactate fermentation. Despite previous detection of acetone and isopropanol in PPR wetland pore fluids (11), none of the MAGs reconstructed here contained the minimal set of genes (see Materials and Methods) to determine potential for acetone/isopropanol-butanol-ethanol fermentation.

Only one *Chloroflexi* MAG (metabat2.725) encoded a glyceraldehyde-3-phosphate:ferredoxin oxidoreductase; therefore, we infer that the conversion of glucose to pyruvate does not proceed as in P. furiosus (20), but as in the conventional EMP glycolysis in the majority of the MAGs in this study. From pyruvate (or indolepyruvate, 2-oxoglutarate, and 2-oxoisovalerate), the pathway may involve the reactions described by Ma et al. (21) as displayed in Fig. 1. The PFOR reaction would generate acetyl coenzyme A (acetyl-CoA) and acetaldehyde. Acetyl-CoA would be converted into acetate by ACS, while acetaldehyde would be converted into acetate by AFOR or into ethanol by an alcohol dehydrogenase.

Of the 14 MAGs encoding putative isopropanol dehydrogenases, only 3 also had other genes involved in isopropanol-butanol-ethanol fermentation. The KSB1-affiliated MAG (metabat2.380; ∼95% complete) encoded a putative isopropanol dehydrogenase, phosphotransbutyrylase, and butyrate kinase and represents the most likely microorganism involved in isopropanol-butanol-ethanol fermentation in this study. Despite this, no transcripts for its putative isopropanol dehydrogenases were detected. Similarly, the *Acidobacteria* MAG maxbin2.0013 (∼61% complete) encoded a putative isopropanol dehydrogenase and a butyraldehyde dehydrogenase, while the *Alphaproteobacteria* MAG metabat2.370 (∼30% complete) encoded both acetoacetate decarboxylase and a putative isopropanol dehydrogenase. However, in contrast to the KSB1 MAG, transcripts matching putative isopropanol dehydrogenases were detected in both of these MAGs. Although we acknowledge that genome incompleteness may play a role in the detection of truncated pathways for isopropanol-butanol-ethanol fermentation, we note that even relatively complete genomes (∼95%), such as the *Burkholderiales* MAG metabat2.802 and the *Ignavibacteriae* MAG metabat2.334, lacked other genes in this fermentation pathway aside from a putative isopropanol dehydrogenase, suggesting that these microorganisms may use novel pathways for isopropanol production or consumption. In addition, 5 of the 14 putative isopropanol dehydrogenases that were inferred to be active (via mRNA transcripts) were encoded in *Acidobacteria* MAGs and 2 in *Planctomycetes* MAGs, taxa that are not currently known to play a role in isopropanol cycling. Together, these data indicate that novel isopropanol-producing pathways may await discovery and that phyla that were previously unrecognized in playing a role in isopropanol metabolism may be important in this process in Prairie Pothole wetlands.

Organisms with the ability to cycle alcohols were implicated in reducing a range of oxidized substrates. Seven of the MAGs contained marker genes for sulfate reduction, while 31 were potentially able to catalyze dissimilatory nitrate reduction to ammonium (DNRA) (Fig. 5). Although none of the MAGs contained genes encoding the complete denitrification pathway, 38 contained a truncated pathway. Reflecting the metabolic versatility of these microorganisms, the majority (54 of 62) of MAGs were also predicated to perform oxygen reduction. Only 2 MAGs did not have potential for any of these respiratory processes. *Thaumarchaeota* maxbin2.0428 had no NADH dehydrogenase or cytochrome *c* reductase and lacked a complete TCA cycle. Both *Thaumarchaeota* MAGs had *amoBC* but not *amoA*. *Actinobacteria* maxbin2.0055 contained an NADH dehydrogenase but lacked a TCA cycle or cytochrome *c* reductase; however, given that the genome was only ∼50% complete, additional undetected respiratory terminal reductases may be affiliated with this microorganism (Table S1).

## DISCUSSION

This study aimed to investigate potential microbial genes, taxa, and pathways implicated in the production of ethanol and isopropanol in Prairie Pothole wetlands, since these alcohols have been previously measured in millimolar concentrations in sediment pore waters (11). MAGs were obtained from sediment samples in which these alcohols were measured and screened for alcohol metabolism genes, resulting in the selection of 62 MAGs for further analyses in this study.

Of 16 phylum-level groups, the potential for ethanol production was detected in 13, while candidate isopropanol dehydrogenases were identified in 8 (see the supplemental material). This potential for isopropanol metabolism should be considered putative, given that enzymatic studies are needed to confirm substrate specificity of these alcohol dehydrogenases. Most of the MAGs missing the minimal criteria for a fermentative pathway still had genes commonly involved in fermentation (e.g., lactate dehydrogenase, phosphotransacetylase, acetate kinase, and formate dehydrogenase). The reconstruction of MAGs from complex sedimentary matrices is an ongoing computational challenge, with the result that only 28 of the genomes analyzed here were more than 70% complete. Genome incompleteness may therefore explain the absence of a complete pathway for acetone/isopropanol-butanol-ethanol fermentation in these genomes, despite the detection of genes (or subunits), including acetoacetyl-CoA:acetate/butyrate CoA transferase, acetoacetate decarboxylase, putative isopropanol dehydrogenase, butyraldehyde dehydrogenase, butanol dehydrogenase, phosphotransbutyrylase, and butyrate kinase in 30 MAGs, with some genomes having up to 3 of these genes (Table S1). While we acknowledge the potential underestimation of microbial groups implicated in alcohol cycling in Prairie Pothole wetlands, we argue that the MAGs selected for this study had metabolic potential, abundances, and transcript activity that support their role in alcohol cycling in this ecosystem.

The clustering of 167 MAG-encoded ADH sequences from this study with reference sequences allowed the inference of ADH substrates. However, a greater challenge was determining the potential for alcohol production versus alcohol consumption based on genomic data alone. While some alcohol dehydrogenases run preferentially in the oxidative or fermentative direction, many are reversible and utilize a broad range of substrates (22–24). Therefore, we cannot rule out that many of these microorganisms may utilize ethanol and/or isopropanol as electron donors. While ethanol oxidation enters central carbon metabolism via acetyl-CoA, isopropanol oxidation is less understood and is hypothesized to follow the order isopropanol → acetone → acetol → methylglyoxal → pyruvate in unclassified bacteria isolated from environmental samples (25).

Since the first report on isopropanol dehydrogenase activity in photosynthetic *Rhodopseudomonas* species in 1940 (26) and the confirmation that the enzyme generated acetone by direct dehydrogenation of isopropanol (27), a variety of studies have demonstrated the microbial ability to oxidize isopropanol. Hoshino reported this metabolism in Lactobacillus brevis, which expressed an enzyme running preferentially in the oxidation direction (28). Interestingly, methylotrophic *Bacillus* strains have been shown to oxidize not only methanol, but also ethanol, isopropanol, *n*-propanol, isobutanol, *n*-butanol, and a variety of methylated amines, sugars, and organic acids (29). Moreover, it is known that methanogens can utilize isopropanol as a hydrogen donor (30), generating acetone via F420-dependent alcohol dehydrogenase, and that acetone can be fermented to acetate by sulfate-reducing bacteria (31) or degraded by nitrate (32) and sulfate reducers (33) via different pathways (acetone decarboxylase in the first and 2-hydroxyisobutyryl-CoA mutase and 3-hydroxybutyryl-CoA dehydrogenase in the latter). Further research is required to elucidate the complex metabolic networks in which the putative isopropanol dehydrogenases identified in this study participate, as well as whether they act as isopropanol dehydrogenases.

Interestingly, efforts in bioengineering P. furiosus for industrial-scale ethanol production have focused on the insertion of a heterologous acetaldehyde-utilizing alcohol dehydrogenase (AdhA) and a carbon monoxide dehydrogenase (34) and also in deleting the aldehyde:ferredoxin oxidoreductase while expressing a heterologous bifunctional acetaldehyde/alcohol dehydrogenase (35). The first approach resulted in an ethanol yield of 20 mM. In Prairie Pothole wetland sediments, natural ethanol concentrations reach 4 mM, and MAGs with potential for P. furiosus-like fermentation encoded the genomic variations targeted in bioengineering. Moreover, the *Rhodobacter* nitrogen fixation (Rnf) complex was present in some of these MAGs and could be utilized to oxidize ferredoxin and reduce NAD^+^ while pumping sodium ions, subsequently utilized for ATP synthesis. Concomitant NADH formation by this Rnf complex could be coupled to alcohol production by NAD-dependent ADHs. While laboratory isolation and biochemical studies are required to test these hypotheses, preliminary genomic data suggest that Prairie Pothole wetlands could be attractive sources of novel microorganisms for the industrial production of alcohols.

Given the diversity of energetically favorable respiratory metabolisms encoded within these genomes, would fermentative processes be expected in PPR sediments? We hypothesize that the heterogeneous sediment matrix allows the formation of anoxic or hypoxic pockets where electron acceptors are temporarily depleted, creating the conditions required for fermentation to occur (36). As geochemical conditions dynamically shift (e.g., sulfate upward influx from pyrite oxidation as oxygenated groundwater flows through the bedrock, nitrogen inputs from agricultural runoff during storm events, or temporary oxygenation of sediments at the water-sediment interface from wind-driven water column mixing) microorganisms may return to respiratory processes or enter into a fermentative mode. Dalcin Martins et al. investigated the genomes of candidate sulfate-reducing bacteria in PPR wetland sediments and similarly observed the potential for other respiratory processes: DNRA, denitrification, and oxygen reduction (13). Despite this metabolic flexibility, the extremely high measured SRRs indicate that sulfate reduction was still a critical process contributing to carbon mineralization in these sediments. We suggest that the putative fermenters identified here show similar metabolic flexibility to the candidate SRB in PPR sediments. Indeed, ∼65% of binned ADHs were transcribed, indicating the selected MAGs represent microorganisms active in alcohol metabolism. Via this metabolic flexibility, redox chemistry is able to deviate from expected reaction order, as has been observed in other studies (1, 11, 37–39). For example, sulfate reducers may preferentially oxidize ethanol over acetate due to higher thermodynamic yields (40). Thus, the incomplete oxidation of ethanol to acetate may provide additional substrate for acetoclastic methanogens, leading to cooperation instead of competition. Moreover, high substrate concentrations may alleviate thermodynamic inhibition and allow sulfate reducers and methanogens to coexist. High organic carbon loads have been previously hypothesized to allow for the cooccurrence of acetoclastic methanogens and sulfate reducers in coastal marine sediments (41). Alternatively, coculture experiments indicated that H_2_ leaking from acetoclastic methanogens could support sulfate reducers (42), while methanogens may induce sulfate reducers to enter a fermentative mode (43). Here, seven MAGs presented potential for sulfate reduction and also fermentation, indicating that the latter is also a potential explanation—among many—for the cooccurring high methane emissions and sulfate reduction rates measured in Prairie Pothole wetlands.

We conclude that PPR wetland sediments harbor a vast diversity of candidate alcohol-cycling microorganisms encoding a variety of alcohol dehydrogenases with potential for unusual, classical, and novel fermentation pathways. We have been able to assign putative substrates to alcohol dehydrogenases and better understand alcohol production in this ecosystem, which is predicted to directly support the highest sulfate reduction rates ever reported, and indirectly, via fermentation—or directly, via F420-dependent alcohol dehydrogenases—support methanogenesis that results in extremely high methane emissions (11, 44, 45). Alcohols are likely key intermediates in sediment carbon cycling and in CO_2_ and CH_4_ generation, highlighting the need for systematic measurements in sediment pore waters, as well as isolation and biochemical investigations of key microorganisms implicated in alcohol cycling. Particularly, isopropanol metabolism in natural environments requires more attention, given both the industrial importance of this alcohol and the potential role as an intermediate in carbon cycling in sedimentary systems. The roles of the putative isopropanol dehydrogenases identified in this study remain to be elucidated.

## MATERIALS AND METHODS

### Sample collection, DNA extraction and sequencing, metagenome assembly, and binning.

Sediment core samples were collected from two adjacent wetlands, P7 and P8, at the U.S. Geological Survey-managed Cottonwood Lake Study Area near Jamestown, ND (11). Accordingly, samples spanned wetland type (P7 and P8), season (winter, spring, and summer), and depth (1 to 3, 10 to 12, and 19 to 21 cm). The 18 sediment samples chosen for metagenomic sequencing are the same samples previously analyzed (see supplemental file 1 in reference 13), and MAGs selected for this study belong to this same MAG data set (13). As such, five previously published MAGs have been reanalyzed and included in this study: maxbin2.0908, maxbin2.1011, maxbin2.0177, metabat2.783, and metabat2.793. All 57 other MAGs were analyzed in the present study.

DNA was extracted using the MoBio PowerLyzer Powersoil DNA isolation kit (Mo Bio Laboratories, Inc., Carlsbad, CA) and quantified using a Qubit fluorometer (Invitrogen, Carlsbad, CA). Metagenomic sequencing was performed at the DOE Joint Genome Institute. Briefly, libraries were constructed with an Illumina regular fragment (300 bp) in tubes. For this, 100 ng of DNA was sheared to 300 bp using the Covaris LE220 and size selected using SPRI beads (Beckman Coulter, Brea, CA). The fragments were treated with end-repair, A-tailing, and ligation of Illumina-compatible adapters (Integrated Device Technology, San Jose, CA) using a KAPA-Illumina library creation kit (KAPA Biosystems, Wilmington, MA). The prepared libraries were quantified using KAPA Biosystems' next-generation sequencing library qPCR kit and run on a Roche LightCycler 480 real-time PCR instrument. The quantified library was then multiplexed with other libraries, and a pool of libraries was then prepared for sequencing on the Illumina HiSeq sequencing platform utilizing a TruSeq paired-end cluster kit, v4, and Illumina’s cBot instrument to generate a clustered flow cell for sequencing. Sequencing of the flow cell was performed on the Illumina HiSeq2500 sequencer using HiSeq TruSeq SBS sequencing kits, v4, following a 2 × 150 indexed run recipe.

After read trimming and quality control using BBDuk and BBMap (46) as previously described (13), metagenome assembly was performed using MEGAHIT v1.0.3 (47) using a range of kmers at default settings. The 18 assemblies were merged using the first part of the MeGAMerge pipeline (48) with default parameters. Only contigs larger than 1,500 bp were retained. Reads were mapped back to the final contig set using Bowtie2 (49). The generated sequence mapping files were handled and converted as needed using SAMtools 1.6 (50). Metagenome binning into draft genomes was performed using three different binning algorithms with default parameters: CONCOCT 0.4.1 (51), MaxBin2 v2.2.3 (52), and MetaBAT2 v2.10.2 (53). The three resulting bin sets were supplied to DAS Tool 1.0 (54) for consensus binning and dereplication, generating an optimized set of MAGs. A single-copy marker gene analysis was performed using CheckM 1.0.7 (55) to assess MAG quality.

### Annotation and gene analyses.

Contigs (contiguous DNA sequences) were gene called and annotated using an in-house annotation pipeline as previously described (56, 57). Briefly, genes were called with Prodigal (58) and annotated based on forward and reverse blast hits (using a minimum 300-bit score threshold for reciprocal matches and 60-bit score threshold for one-way matches) to amino acid sequences in the databases UniRef90 and KEGG, while motifs were analyzed using InterProScan.

MAGs were selected for in-depth gene analyses based on the potential for alcohol cycling. At least 110 genes were searched for in each draft genome, totaling ∼8,000 genes (see Table S1 in the supplemental material) involved in a variety of pathways. The minimal criteria to determine whether a MAG had the potential for a pathway are presented in Table 1. The abundance of MAGs was inferred from total normalized coverage (59), calculated as the total base pairs of mapped reads (summed across all 18 metagenomes) multiplied by 1 Gbp divided by genome length and metagenome base pairs (summed across all 18 metagenomes).

**TABLE 1:** Minimal criteria to determine the potential for a pathway, process, or enzyme

Metabolic trait	Criteria used to determine metabolic potential
Sugar utilization	At least one sugar-specific phosphotransferase component II enzyme or sugar kinase
EMP glycolysis	Six of ten genes (or five, with one being a phosphofructokinase)
Pentose phosphate pathway	Four of seven genes
Entner-Doudoroff pathway	Both 6-phosphogluconate dehydratase and 2-keto-3-deoxy-6-phosphate-gluconate aldolase
Pyruvate or 2-oxoglutarate dehydrogenase complex	At least component E1 or component E2
TCA cycle	Five of nine genes; if 2-oxoglutarate dehydrogenase was missing but 2-oxoglutarate:ferredoxin oxidoreductase was present, it counted as an alternative; if both were missing, the TCA cycle was considered incomplete; for succinate dehydrogenase/fumarate reductase, at least two of four subunits needed to be present
Pyrococcus furiosus-like fermentation	Pyruvate, indolepyruvate, 2-oxoisovalerate, or 2-oxoglutarate:ferredoxin oxidoreductase, and two of another three components: (i) acetyl-CoA synthetase; (ii) aldehyde:ferredoxin oxidoreductase; and (iii) ferredoxin:NADP^+^ oxidoreductase
Heterofermentative lactate fermentation	Potential for EMP, phosphoketolase, lactate dehydrogenase, aldehyde dehydrogenase, and ADH
Mixed acid fermentation	Pyruvate-formate lyase (PFL) or PFL-activating enzyme (AE), formate dehydrogenase, acetate kinase, potential for TCA or succinate dehydrogenase or lactate dehydrogenase, aldehyde dehydrogenase, and ADH
Butanediol fermentation	Aldehyde dehydrogenase, ADH, and 2,3-butanediol dehydrogenase
Acetone-butanol-ethanol fermentation	Acetoacetyl-CoA: acetate/butyrate CoA transferase, acetoacetate decarboxylase, phosphotransbutyrylase, butyrate kinase, aldehyde dehydrogenase and ADH
Isopropanol-butanol-ethanol fermentation	Acetoacetyl-CoA: acetate/butyrate CoA transferase, acetoacetate decarboxylase, phosphotransbutyrylase, butyrate kinase, aldehyde dehydrogenase, ADH, and isopropanol dehydrogenase
Sulfate reduction	At least one subunit of the dissimilatory sulfide reductase (*dsrABD*) and no sox pathway
DNRA	At least one ammonia-forming nitrite reductase (*nirBD* or *nrfAH*)
Denitrification (partial)	At least one of the following: nitrate reductase (*narGHI* or *napAB*), nitrite reductase (*nirKS*), nitric oxide reductase (*norBC*), or nitrous oxide reductase (*nosZ*)
Oxygen respiration	At least one of the following oxygen reductases: *aa*_3_-type cytochrome *c* oxidase (*coxABCD*), *cbb*_3_-type cytochrome *c* oxidase (*ccoNOPQ*), cytochrome *ba*_3_ heme quinol oxidase, cytochrome *bo*_3_ ubiquinol oxidoreductase (*cyoABCDE*), cytochrome *aa*_3_-600 menaquinol oxidase (*qoxABCD*), or cytochrome *bd*_1_ ubiquinol oxidoreductase/cyanide-insensitive oxidase (*cydABX*)

The taxonomical classification of these selected MAGs was determined based on lineage-specific phylogenetic markers from CheckM (55). To resolve instances in which CheckM could not classify a MAG beyond domain level or to confirm taxonomy, binned *rpsC* sequences (encoding the ribosomal protein S3) were used to place MAGs in a phylogenetic tree containing reference sequences from the 2016 update of the tree of life (60). MAGs including genes for alcohol dehydrogenases were used to build a phylogenetic tree containing also reference sequences retrieved from the National Center for Biotechnology Information (NCBI; minimum sequence length of 100 amino acids for any sequences in this tree). For these phylogenetic trees, entire amino acid sequences were aligned with MUSCLE v3.8.31 (61), and columns with at least 95% gaps were removed using Geneious 9.0.5 (62). Trees were built using FastTree v2.1.10, which infers approximately maximum-likelihood phylogenetic trees (63), using default parameters under the Jones-Taylor-Thornton model of amino acid evolution, and visualized with iToL (64). Figures were edited in Adobe Illustrator v16.0.0 (Adobe Systems, Inc., San Jose, CA).

### Metatranscriptomic analyses.

In total, six sediment samples for metatranscriptomics were sent to the Environmental Molecular Sciences Laboratory (EMSL) in Richland, WA: MayP7_core1_1-3cm, MayP8_core1_1-3cm, SepP7_core6_1-3cm, SepP8_core1_1-3cm, SepP7_core5_10-12, and SepP8_core2_10-12cm. These samples correspond to our previously published data (11, 13). RNA was extracted from sediments using the RNeasy Powersoil Total RNA kit (Qiagen, Hilden, Germany), followed by genomic DNA removal and cleaning using Qiagen’s RNase-Free DNase set kit and an RNeasy minikit. The integrity of the RNA samples was assessed using an Agilent 2100 bioanalyzer (Agilent, Santa Barbara, CA). RNA samples having RNA integrity numbers between 9 and 10 were used in this work. A Ribo-Zero rRNA removal kit plant (Illumina, San Diego, CA) was used for enrichment of transcripts. The SOLiD Total RNA-Seq kit (Thermo Fisher Scientific, Waltham, MA) was used to construct template cDNA for RNA-Seq according to the manufacturer’s instructions. Briefly, mRNA was fragmented using chemical hydrolysis, followed by ligation with strand-specific adapters and reverse transcript to generate cDNA. The cDNA fragments, 150 to 250 bp in size, were isolated and amplified through 15 amplification cycles to produce the required number of templates for the SOLiD EZ Bead system, which was used to generate the template bead library for ligation base sequencing by 5500xl SOLiD instrument (Thermo Fisher Scientific). The 50-base short read sequences produced by the SOLiD sequencer were mapped in color space using the whole-transcriptome analysis pipeline in Life Technologies LifeScope software v2.5 against the metagenome. Adapter and quality trimming and quality control were performed using LifeScope’s default settings. Because LifeScope software does not accommodate the large number of genes present in the metagenome, the 7,471,083 gene sequences were collapsed into 95 artificial chromosomes. A companion gtf file was also created, with gene locations adjusted accordingly. Since the number of annotated genes that LifeScope is designed to handle is also limited, a placeholder gtf was provided for the LifeScope pipeline. Output bam files were then provided as input for htseq-count (65) for mapping of aligned reads to genes, with the “nonunique” argument set to “all.” LifeScope selects locations based on the best read-matching score and randomly chooses locations when multiple loci receive the same score. The “–nonunique all” setting of htseq-count allows these reads to be included. RPKM (reads per kilobase per million mapped reads) values for each gene were calculated as the number of mapped reads times 10^9^ divided by the total number of reads in that sample times the gene length in base pairs using R software (66).

### Data availability.

Raw reads, trimmed reads, individual assemblies, and quality control reports are available at the JGI genome portal under project name “Seasonal Sulfur Cycling as a Control on Methane Flux in Carbon-Rich Prairie Pothole Sediment Ecosystems” and JGI proposal ID 2025 (https://genome.jgi.doe.gov/portal/Seasulecosystems/Seasulecosystems.info.html). All MAGs used in this study and metatranscriptomic data were deposited on NCBI under BioProject number PRJNA330672. Additional files are publicly available at CyVerse (https://de.cyverse.org/de/): amino acid fasta files, annotation files, ADH sequences, and trees from this study. To access these files, users must create an account, log in, and enter the folder pathway “/iplant/home/pdalcin/PPR_alcohol_files.” The merged contig set from Dalcin Martins et al. (13) is available at “/iplant/home/pdalcin/microbiome_files.”

## Supplementary Material

Supplemental file 1

Supplemental file 2

Supplemental file 3

Supplemental file 4
