# Supplementary material for: Wetland Sediments Host Diverse Microbial Taxa Capable of Cycling Alcohols
Source: Appl Environ Microbiol. 2019 May 30;85(12):e00189-19. doi: 10.1128/AEM.00189-19 (PMC6544822; doi:10.1128/AEM.00189-19)
Supplement: Supplemental file 1 [file AEM.00189-19-s0004.pdf]

Supplemental Text

Wetland sediments host diverse microbial taxa capable of cycling alcohols

Paula Dalcin Martins[1], Jeroen Frank[1], Hugh Mitchell[2], Lye Meng Markillie[2], Michael J. Wilkins[3*]
[1]Department of Microbiology, Radboud University Nijmegen, The Netherlands
[2]Pacific Northwest National Laboratory, Richland, WA, USA
[3]Department of Soil and Crop Sciences, Colorado State University, CO, USA

*Corresponding author's email: Mike.Wilkins@ColoState.edu

Metabolic characterization of MAGs

Two *Thaumarchaeota* MAGs (maxbin2.0118 and maxbin2.0428) were identified in this study and shared most traits: both had an incomplete tricarboxylic acid (TCA) cycle, and were lacking genes for Embden–Meyerhof–Parnas (EMP) glycolysis, pyruvate dehydrogenase (PDH) complex, and the investigated fermentative pathways. However, maxbin2.0118 contained a putative isopropanol dehydrogenase for which transcripts were detected, and genes encoding the pentose phosphate pathway (PPP), NADH dehydrogenase, a cytochrome c reductase, and partial denitrification genes *nirK* and *nosZ*. Meanwhile, maxbin2.0428 did not encode any electron transport chain and terminal reductase genes. Despite lacking the PPP, it encoded a ribokinase, F420-dependent glucose dehydrogenase, transketolase and transaldolase (Supplemental Table 1). Recently, the potential for amino acid fermentation via indolepyruvate:ferredoxin oxidoreductase (IFOR) has been identified in the ammonia-oxidizing *Thaumarchaeota* organism *Candidatus* Nitrosocaldus islandicus (1), suggesting that members of this phylum may be more metabolically versatile than currently appreciated. Nonetheless, the prevalence of dissimilatory nitrate reduction to ammonium (DNRA) potential in our MAGs (31 out of 62) suggests that abundant ammonia could be formed in PPR wetland sediments, providing substrate for such metabolism. Further studies are required to elucidate the role of aldehyde and alcohol dehydrogenases in these PPR *Thaumarchaetota* organisms, particularly given their reported abundance in soils and sediments (2–5).

The potential for butyrate and butanol production was identified in MAG metabat2.451, which was ~86% complete and tentatively assigned to the phylum *Calditrichaeota* due to its S3 ribosomal protein forming a monophyletic group with *Caldithrix*. The MAG encoded a phosphotransbutyrylase, butyrate kinase, and butanol dehydrogenase, in addition to phosphotransferase system enzyme II components specific for mannose, fructose, and cellobiose, and sugar kinases specific for glucose, rhamnulose, galactose, L-ribulose, xylulose, and fructose. Moreover, the MAG encoded a full RNF complex (*rnfABCDGE*) and the potential for EMP glycolysis, PPP, PDH complex *P. furiosus*-like fermentation with both PFOR and IFOR, as well as heterofermentative lactate fermentation and oxygen respiration via *cydAB*. These metabolic traits have some similarities and differences to *Caldithrix abyssi*, which was isolated as a pyruvate and amino acid fermenter, while also performing nitrate reduction with hydrogen and acetate as electron donors (6). Genome sequencing of this isolate revealed multiple glycoside hydrolases which enable *C. abyssi* to grow on starch, cellobiose, glucomannan, and xyloglucan (7), while screening of additional *Calditrichaeota* genomes have revealed potential for amino acid fermentation and oxygen reduction (8).

The potential for *P. furiosus*-like fermentation with PFOR, 2-oxoglutarate:ferredoxin oxidoreductase (OGFOR), and 2-oxoisovalerate:ferredoxin oxidoreductase (OIFOR) was also detected in the KSB1-affiliated MAG metabat2.380. The ability to perform DNRA (*nrfAH*), and oxygen respiration (NADH dehydrogenase and *cydA*) was additionally inferred from the genome. The MAG encoded 2 putative isopropanol dehydrogenases, neither of which were detected in metatranscriptomic data, a phosphotransbutyrylase, and a butyrate kinase. Although the minimal criteria for isopropanol-butanol-ethanol fermentation were not met, the MAG also encoded hydroxybutyryl-CoA dehydrogenase, enoyl-CoA hydratase, and butyryl-CoA dehydrogenase. KSB1 MAGs have been previously suggested to play a role in organic carbon degradation and fermentation based on high numbers of genes involved in hydrolysis of organic carbon and encoding for extracellular peptidases (9). Some MAGs also encode the complete β-oxidation pathway and genes for anaerobic hydrocarbon degradation, as well as nitrate reductases (napA) (10). Ethanol was suggested as the electron donor for denitrification in these MAGs. Therefore, our results expand the metabolic diversity of KSB1 and highlight the need for the elucidation of alcohol metabolism in this phylum.

When first proposed as a candidate phylum, *Rokubacteria* were predicted to be acetoclastic heterotrophs likely also utilizing beta-oxidation of fatty acids for energy. These first genomes encoded a single-chain PFOR and a complete pathway for butyrate metabolism but missed several key glycolysis genes, while also encoding the potential for sulfur oxidation and nitrate reduction (11). Our *Rokubacteria* MAG (metabat2.850) was distinct in that it had potential for complete EMP glycolysis (missing only the glucose-6-phosphate isomerase), *P. furiosus*-like fermentation with PFOR, IFOR, and OGFOR, heterofermentative lactate fermentation, and mixed acid fermentation. It also encoded the potential for sulfur oxidation (*soxFYZ*), DNRA (*nrfA*), partial denitrification (*narGH* and *norBC*) and oxygen respiration (NADH dehydrogenase, cytochrome c reductase, aa_3-type cytochrome c oxidase subunits *coxABC*, cbb_3-type cytochrome c oxidase subunits *ccoNOP*, cytochrome bo_3 ubiquinol oxidoreductase subunit *cyoC,* and cytochrome bd_I ubiquinol oxidoreductase subunits *cydAB*). Moreover, our *Rokubacteria* MAG encoded a putative isopropanol dehydrogenase for which transcripts were detected. A recent analysis of 52 *Rokubacteria* MAGs revealed potential for hydrocarbon oxidation (including methane oxidation via pMMO genes), oxygen, nitrate and sulfate reduction, and production of acetate and ethanol (12). Together, these results expand the inferred metabolic versatility in this phylum.

Latescibacteria were initially described as obligate fermenters due to the absence of an electron transport chain and were predicted to generate acetate, succinate, propanol and propionate (13, 14). However, more recent analyses revealed that members of this group could additionally generate lactate, acetoin, and ethanol, and also respire oxygen (15). Moreover, a recent study used quantitative DNA stable isotope probing to link *Latescibacteria* to organic carbon turnover in lacustrine sediments (16). Our results corroborate these descriptions: the *Latescibacteria* MAG maxbin2.0022 recovered in this study had the potential for EMP glycolysis, PPP, the PDH complex, TCA cycle, mixed acid fermentation and oxygen respiration (NADH dehydrogenase, cytochrome c reductase, aa_3-type cytochrome c oxidase subunits *coxABCD*, and cytochrome bd_I ubiquinol oxidoreductase subunits *cydA*). However, this MAG adds the potential for *P. furiosus*-like fermentation with PFOR, OGFOR, and OIFOR, and for butanol and butyrate production given that it encoded a butyraldehyde dehydrogenase, phosphotransbutyrylase, and butyrate kinase.

Cyanobacteria are capable of homo- and heterofermentative lactate fermentation, mixed acid fermentation and homoacetate fermentation (17), and have been reported as the dominant

fermenters in microbial mats, providing acetate for sulfate-reducing bacteria and *Chloroflexi* (18). Our *Cyanobacteria* MAG metabat2.561 had potential for heterofermentative lactate fermentation and oxygen respiration (NADH dehydrogenase, cytochrome c reductase, *coxBC* and *cydB*). Also, this draft genome encoded genes for photosynthesis (some subunits of photosystem I and II, and cytochrome b6/f complex).

Spring and collaborators isolated 5 strains of *Planctomycetes* from lake and saline sediments that encoded the RNF complex and were strict fermenters, producing ethanol via mixed acid fermentation (19). This supports the potential role of our *Planctomycetes* MAGs in alcohol production in PPR wetland sediments. Of four *Planctomycetes* MAGs in this study (metabat2.867, maxbin2.1320, maxbin2.1616, and metabat2.703), 2 had potential for *P. furiosus*-like fermentation, and all encoded potential for respiratory processes. One MAG (maxbin2.1616) encoded the acetoacetyl-CoA: acetate/butyrate CoA transferase subunit *atoD*, and another (metabat2.867) encoded 2 putative isopropanol dehydrogenases, both detected in metatranscriptomic data.

The MAG metabat2.423 formed a monophyletic group with a *Zetaproteobacteria* sequence in the S3 ribosomal protein tree (Figure 1) and had potential for EMP glycolysis, PPP, PDH complex, TCA cycle, *P. furiosus*-like fermentation (PFOR, IFOR, and OGFOR), partial denitrification (*norBC*) and oxygen reduction (NADH dehydrogenase, cytochrome c reductase, *coxBC* and *cydAB*). The MAG also encoded the acetoacetyl-CoA: acetate/butyrate CoA transferase subunit *ydiF*, but no other genes involved in acetone or isopropanol-butanol-ethanol fermentation. *Zetaproteobacteria* members have been assigned roles in iron oxidation (20–23); however, no study had previously implicated these microorganisms in alcohol cycling.

Ignavibacteriae isolates were capable of fermenting a variety of sugars and performing aerobic and anaerobic respirations in previous studies (24, 25). In line with these results, of five *Ignavibacteriae* MAGs (maxbin2.1368, metabat2.211, metabat2.334, maxbin2.1403, and maxbin2.1615), 3 had potential for *P. furiosus*-like fermentation (all had at least PFOR and OGFOR), 1 was putatively able to perform heterofermentative lactate fermentation, and 3 encoded potential for mixed acid fermentation. All encoded genes for respiratory processes. The MAG metabat2.334 encoded a putative isopropanol dehydrogenase for which transcripts were detected.

Proteobacteria, *Acidobacteria*, *Actinobacteria*, *Bacteroidetes*, *Spirochaetes* and *Verrucomicrobia* have commonly been highlighted as key fermenters in peatlands, being implicated in the degradation of cellobiose, cellulose, glucose, and xylose and reported to impact the ratio of CO_2/CH_4 in greenhouse gas emissions and provide substrates for methanogenesis (26–29). These observations support the alcohol cycling roles tentatively assigned to the remaining groups identified in this study. Of six *Acidobacteria* MAGs (maxbin2.0065, metabat2.299, metabat2.166, maxbin2.0013, metabat2.851, and metabat2.341), 3 had the potential for *P. furiosus*-like fermentation, 2 for heterofermentative lactate fermentation, and 2 for mixed acid fermentation. Interestingly, 4 MAGs had putative isopropanol dehydrogenases identified in metatranscriptomic data (maxbin2.0013, metabat2.166, metabat2.299, and metabat2.851). With the exception of metabat2.851, these MAGs encoded a variety of sugar kinases (glucose, galactose, xylulose, ribose, fructose, fucose, rhamnulose, L-ribulose kinase).

Of four *Actinobacteria* MAGs (maxbin2.0055, metabat2.191, metabat2.143, and metabat2.324), only metabat2.143 had potential for *P. furiosus*-like fermentation (PFOR, IFOR, OGFOR) and

DNRA and encoded the acetoacetyl-CoA: acetate/butyrate CoA transferase subunit *atoD* and a butyrate kinase. All MAGs encoded PFOR and ACS. MAG maxbin2.0055 encoded an acetoacetate decarboxylase but no isopropanol dehydrogenase.

The two *Bacteroidetes* MAGs (maxbin2.1578 and the most complete draft genome from this MAG set, metabat2.99) encoded the RNF complex. MAG metabat2.99 also encoded *coxABCD* and had potential for *P. furiosus*-like fermentation (PFOR, IFOR, and OGFOR), heterofermentative lactate fermentation and mixed acid fermentation. Interestingly, maxbin2.1578 encoded a phosphotransbutyrylase, and both MAGs encoded a butyrate kinase.

Of eight *Chloroflexi* draft genomes (maxbin2.1011, metabat2.402, metabat2.178, maxbin2.0908, metabat2.725, maxbin2.0031, metabat2.854, and maxbin2.0642), 6 had potential for *P. furiosus*-like fermentation, and 1 for mixed acid fermentation. All had potential for respiratory processes. One MAG encoded the acetoacetyl-CoA: acetate/butyrate CoA transferase subunit *atoD*, one MAG encoded an acetoacetate decarboxylase, and two encoded putative isopropanol dehydrogenases (one was detected in metatranscriptomic data); however, those were 4 different MAGs. A fifth MAG (metabat2.178) encoded a phosphotransbutyrylase, butyrate kinase and butanol dehydrogenase. Remarkably, a variety of sugar kinases were encoded – glucose, fructose, xylulose, galactose, rhamnulose, L-ribulose, ribose, sedoheptulose, and fucose kinase.

While Hug and collaborators have suggested that *Chloroflexi* play roles in sediment carbon cycling including fermentation (30), Baker and collaborators have reported fermentation potential in members of the *Chloroflexi*, *Bacteroidetes*, *Plancomycetes*, *Nitrospirae*, and *Spirochaetes* from river sediments, noticing that denitrification genes were distributed among different members (9), as observed in this and other studies (31). Saad and collaborators enriched sulfate-reducing and fermentative communities from intertidal sediments and transiently exposed the cultures to oxygen or nitrate (32). Remarkably, sulfate reducers coexisted with facultative nitrate reducers or aerobes, and exposure to oxygen and nitrate did not suppress fermentation or sulfate reduction. In their study, the identified fermenters included members of the phyla *Fermentibacteria*, *Firmicutes*, *Spirochaetes*, and *Chloroflexi*.

The *Nitrospirae* MAG metabat2.72 had potential for EMP glycolysis, PPP, the TCA cycle, *P. furiosus*-like fermentation (PFOR and OGFOR), mixed acid fermentation, and partial denitrification (NADH dehydrogenase and *narGHI*).

Despite not meeting the minimal criteria for any fermentative pathways, all 3 *Alphaproteobacteria* MAGs (maxbin2.0024, metabat2.590, and metabat2.370) encoded IFOR, while 2 encoded PFOR. MAG metabat2.370, which was classified up to family level (*Hyphomicrobiaceae*), was the only MAG in this study to encode both acetoacetate decarboxylase and a putative isopropanol dehydrogenase, which was detected in metatranscriptomic data.

Of four *Betaproteobacteria* MAGs (concoct.173, metabat2.25, metabat2.802, and metabat2.76), 1 had potential for *P. furiosus*-like fermentation, 1 for heterofermentative lactate fermentation, and 2 for mixed acid fermentation. All MAGs had potential for respiratory processes. One MAG encoded an acetoacetate decarboxylase, and another encoded a putative isopropanol dehydrogenase for which transcripts were detected.

Of seven *Deltaproteobacteria* MAGs (maxbin2.0177, metabat2.783, maxbin2.1429, metabat2.793, metabat2.715, metabat2.124, and metabat2.71), 5 had potential for *P. furiosus*-like fermentation, 4 for mixed acid fermentation, and 1 for butanediol fermentation (*Geobacter* metabat2.715, the only MAG encoding the 2,3-butanediol dehydrogenase). All 7 MAGs had potential for respiratory processes. Interestingly, 3 MAGs encoded the acetoacetyl-CoA: acetate/butyrate CoA transferase subunit *atoD*; one of these also encoded subunit *ydiF*.

Of five *Gammaproteobacteria* MAGs (metabat2.308, metabat2.373, metabat2.256, maxbin2.1823, and metabat2.764), 1 had potential for *P. furiosus*-like fermentation, 3 for heretofermentative lactate fermentation, 1 for mixed acid fermentation, and all for respiratory processes. One MAG encoded the acetoacetyl-CoA: acetate/butyrate CoA transferase subunit *atoD*.

The *Spirochaetes* MAG maxbin2.0195 did not meet the minimal criteria for TCA cycle or any fermentative pathways. However, it encoded PFOR, IFOR and OGFOR, acetate kinase and lactate dehydrogenase, a butanol dehydrogenase, as well as the RNF complex and *nrfAH*.

Finally, the *Verrucomicrobia* MAG concoct.366 had potential for DNRA (*nrfAH*), partial denitrification (*nosZ*), and oxygen respiration (NADH dehydrogenase, *coxB* and *ccoNOP*). While it did not meet the minimal criteria for fermentative pathways, it encoded PFOR, acetate kinase, lactate dehydrogenase, formate dehydrogenase, and a butyraldehyde dehydrogenase.

References

1. Daebeler A, Herbold CW, Vierheilig J, Sedlacek CJ, Pjevac P, Albertsen M, Kirkegaard RH, de la Torre JR, Daims H, Wagner M. 2018. Cultivation and genomic analysis of "Candidatus Nitrosocaldus islandicus," an obligately thermophilic, ammonia-oxidizing thaumarchaeon from a hot spring biofilm in Graendalur valley, Iceland. Front Microbiol 9.
2. Ochsenreiter T, Selezi D, Quaiser A, Bonch-Osmolovskaya L, Schleper C. 2003. Diversity and abundance of Crenarchaeota in terrestrial habitats studied by 16S RNA surveys and real time PCR. Environ Microbiol 5:787–797.
3. Lehtovirta LE, Prosser JI, Nicol GW. 2009. Soil pH regulates the abundance and diversity of Group 1.1c Crenarchaeota. FEMS Microbiol Ecol 70:367–376.
4. Fan X, Xing P. 2016. Differences in the composition of archaeal communities in sediments from contrasting zones of Lake Taihu. Front Microbiol 7.
5. Mendes LW, Taketani RG, Navarrete AA, Tsai SM. 2012. Shifts in phylogenetic diversity of archaeal communities in mangrove sediments at different sites and depths in southeastern Brazil. Res Microbiol 163:366–377.
6. Miroshnichenko ML, Kostrikina NA, Chernyh NA, Pimenov N V., Tourova TP, Antipov AN, Spring S, Stackebrandt E, Bonch-Osmolovskaya EA. 2003. Caldithrix abyssi gen. nov., sp. nov., a nitrate-reducing, thermophilic, anaerobic bacterium isolated from a Mid-Atlantic ridge hydrothermal vent, represents a novel bacterial lineage. Int J Syst Evol Microbiol 53:323–329.
7. Kublanov I V., Sigalova OM, Gavrilov SN, Lebedinsky A V., Rinke C, Kovaleva O, Chernyh NA, Ivanova N, Daum C, Reddy TBK, Klenk HP, Spring S, Göker M, Reva ON, Miroshnichenko ML, Kyrpides NC, Woyke T, Gelfand MS, Bonch-Osmolovskaya EA. 2017. Genomic analysis of Caldithrix abyssi, the thermophilic anaerobic bacterium of the novel bacterial phylum Calditrichaeota. Front Microbiol 8.

8. Marshall IPG, Starnawski P, Cupit C, Fernández Cáceres E, Ettema TJG, Schramm A, Kjeldsen KU. 2017. The novel bacterial phylum Calditrichaeota is diverse, widespread and abundant in marine sediments and has the capacity to degrade detrital proteins. Environ Microbiol Rep 9:397–403.
9. Baker BJ, Lazar CS, Teske AP, Dick GJ. 2015. Genomic resolution of linkages in carbon, nitrogen, and sulfur cycling among widespread estuary sediment bacteria. Microbiome 3:14.
10. Dombrowski N, Seitz KW, Teske AP, Baker BJ. 2017. Genomic insights into potential interdependencies in microbial hydrocarbon and nutrient cycling in hydrothermal sediments. Microbiome 5:106.
11. Hug LA, Thomas BC, Sharon I, Brown CT, Sharma R, Hettich RL, Wilkins MJ, Williams KH, Singh A, Banfield JF. 2016. Critical biogeochemical functions in the subsurface are associated with bacteria from new phyla and little studied lineages. Environ Microbiol 18:159–173.
12. Kroeger ME, Delmont TO, Eren AM, Meyer KM, Guo J, Khan K, Rodrigues JLM, Bohannan BJM, Tringe SG, Borges CD, Tiedje JM, Tsai SM, Nüsslein K. 2018. New Biological Insights Into How Deforestation in Amazonia Affects Soil Microbial Communities Using Metagenomics and Metagenome-Assembled Genomes. Front Microbiol 9.
13. Rinke C, Schwientek P, Sczyrba A, Ivanova NN, Anderson IJ, Cheng JF, Darling A, Malfatti S, Swan BK, Gies EA, Dodsworth JA, Hedlund BP, Tsiamis G, Sievert SM, Liu WT, Eisen JA, Hallam SJ, Kyrpides NC, Stepanauskas R, Rubin EM, Hugenholtz P, Woyke T. 2013. Insights into the phylogeny and coding potential of microbial dark matter. Nature 499:431–437.
14. Youssef NH, Farag IF, Rinke C, Hallam SJ, Woyke T, Elshahed MS. 2015. In silico analysis of the metabolic potential and niche specialization of candidate phylum "Latescibacteria" (WS3). PLoS One 10.
15. Farag IF, Youssef NH, Elshahed MS. 2017. Global distribution patterns and pangenomic diversity of the candidate phylum "Latescibacteria" (WS3). Appl Environ Microbiol 83.
16. Coskun ÖK, Pichler M, Vargas S, Gilder S, Orsi WD. 2018. Linking Uncultivated Microbial Populations and Benthic Carbon Turnover by Using Quantitative Stable Isotope Probing. Appl Environ Microbiol 84.
17. Stal LJ, Moezelaar R. 1997. Fermentation in cyanobacteria. FEMS Microbiol Rev.
18. Lee JZ, Burow LC, Woebken D, Craig Everroad R, Kubo MD, Spormann AM, Weber PK, Pett-Ridge J, Bebout BM, Hoehler TM. 2014. Fermentation couples Chloroflexi and sulfate-reducing bacteria to Cyanobacteria in hypersaline microbial mats. Front Microbiol 5.
19. Spring S, Bunk B, Spröer C, Rohde M, Klenk H-P. 2018. Genome biology of a novel lineage of planctomycetes widespread in anoxic aquatic environments. Environ Microbiol.
20. Makita H, Tanaka E, Mitsunobu S, Miyazaki M, Nunoura T, Uematsu K, Takaki Y, Nishi S, Shimamura S, Takai K. 2017. Mariprofundus micogutta sp. nov., a novel iron-oxidizing zetaproteobacterium isolated from a deep-sea hydrothermal field at the Bayonnaise knoll of the Izu-Ogasawara arc, and a description of Mariprofundales ord. nov. and Zetaproteobacteria classis nov. Arch Microbiol 199:335–346.
21. Chiu BK, Kato S, McAllister SM, Field EK, Chan CS. 2017. Novel Pelagic Iron-Oxidizing Zetaproteobacteria from the Chesapeake Bay Oxic–Anoxic Transition Zone. Front Microbiol 8.
22. Laufer K, Nordhoff M, Halama M, Martinez RE, Obst M, Nowak M, Stryhanyuk H,

Richnow HH, Kappler A. 2017. Microaerophilic Fe(II)-Oxidizing Zetaproteobacteria Isolated from Low-Fe Marine Coastal Sediments: Physiology and Composition of Their Twisted Stalks. Appl Environ Microbiol 83.

23. Mori JF, Scott JJ, Hager KW, Moyer CL, Küsel K, Emerson D. 2017. Physiological and ecological implications of an iron- or hydrogen-oxidizing member of the Zetaproteobacteria, Ghiorsea bivora, gen. nov., sp. nov. ISME J 11:2624–2636.
24. Podosokorskaya OA, Kadnikov V V., Gavrilov SN, Mardanov A V., Merkel AY, Karnachuk O V., Ravin N V., Bonch-Osmolovskaya EA, Kublanov I V. 2013. Characterization of *Melioribacter roseus* gen. nov., sp. nov., a novel facultatively anaerobic thermophilic cellulolytic bacterium from the class *Ignavibacteria*, and a proposal of a novel bacterial phylum *Ignavibacteriae*. Environ Microbiol 15:1759–1771.
25. Iino T, Mori K, Uchino Y, Nakagawa T, Harayama S, Suzuki KI. 2010. Ignavibacterium album gen. nov., sp. nov., a moderately thermophilic anaerobic bacterium isolated from microbial mats at a terrestrial hot spring and proposal of Ignavibacteria classis nov., for a novel lineage at the periphery of green sulfur bacteria. Int J Syst Evol Microbiol 60:1376–1382.
26. Schmidt O, Hink L, Horn MA, Drake HL. 2016. Peat: Home to novel syntrophic species that feed acetate- and hydrogen-scavenging methanogens. ISME J 10:1954–1966.
27. Tveit AT, Urich T, Frenzel P, Svenning MM. 2015. Metabolic and trophic interactions modulate methane production by Arctic peat microbiota in response to warming. Proc Natl Acad Sci 112:E2507–E2516.
28. Juottonen H, Eiler A, Biasi C, Tuittila ES, Yrjälä K, Fritze H. 2017. Distinct anaerobic bacterial consumers of cellobiose-derived carbon in boreal fens with different CO2/CH4production ratios. Appl Environ Microbiol 83.
29. Wüst PK, Horn MA, Drake HL. 2009. Trophic links between fermenters and methanogens in a moderately acidic fen soil. Environ Microbiol 11:1395–1409.
30. Hug LA, Castelle CJ, Wrighton KC, Thomas BC, Sharon I, Frischkorn KR, Williams KH, Tringe SG, Banfield JF. 2013. Community genomic analyses constrain the distribution of metabolic traits across the Chloroflexi phylum and indicate roles in sediment carbon cycling. Microbiome 1.
31. Anantharaman K, Brown CT, Hug LA, Sharon I, Castelle CJ, Probst AJ, Thomas BC, Singh A, Wilkins MJ, Karaoz U, Brodie EL, Williams KH, Hubbard SS, Banfield JF. 2016. Thousands of microbial genomes shed light on interconnected biogeochemical processes in an aquifer system. Nat Commun 7.
32. Saad S, Bhatnagar S, Tegetmeyer HE, Geelhoed JS, Strous M, Ruff SE. 2017. Transient exposure to oxygen or nitrate reveals ecophysiology of fermentative and sulfate-reducing benthic microbial populations. Environ Microbiol 19:4866–4881.
